# Nutrition Management for Critically Ill Adult Patients Requiring Non-Invasive Ventilation: A Scoping Review

**DOI:** 10.3390/nu14071446

**Published:** 2022-03-30

**Authors:** Elizabeth Viner Smith, Emma J. Ridley, Christopher K. Rayner, Lee-anne S. Chapple

**Affiliations:** 1 Adelaide Medical School, Faculty of Health and Medical Sciences, The University of Adelaide, Adelaide, SA 5005, Australia; chris.rayner@adelaide.edu.au (C.K.R.); lee-anne.chapple@adelaide.edu.au (L.S.C); 2 Intensive Care Research Unit, Royal Adelaide Hospital, Adelaide, SA 5000, Australia; 3 Australian and New Zealand Intensive Care Research Centre, Monash University, Melbourne, VIC 3004, Australia; emma.ridley@monash.edu; 4 Nutrition Department, Alfred Health, Melbourne, VIC 3004, Australia; 5 Centre of Research Excellence in Translating Nutritional Science to Good Health, The University of Adelaide, Adelaide, SA 5005, Australia; 6 Department of Gastroenterology and Hepatology, Royal Adelaide Hospital, Adelaide, SA 5000, Australia

**Keywords:** critical illness, intensive care, nutrition, non-invasive ventilation

## Abstract

Nutrition management is a core component of intensive care medicine. Despite the increased use of non-invasive ventilation (NIV) for the critically ill, a paucity of evidence on nutrition management precludes recommendations for clinical practice. A scope of the available literature is required to guide future research on this topic. Database searches of MEDLINE, Embase, Scopus, Web of Science, and Google Scholar were conducted to identify original research articles and available grey literature in English from 1 January 1990 to 17 November 2021 that included adult patients (≥16 years) receiving NIV within an Intensive Care Unit. Data were extracted on: study design, aim, population, nutrition concept, context (ICU type, NIV: use, duration, interface), and outcomes. Of 1730 articles, 16 met eligibility criteria. Articles primarily included single-centre, prospective, observational studies with only 3 randomised controlled trials. Key concepts included route of nutrition (*n* = 7), nutrition intake (*n* = 4), energy expenditure (*n* = 2), nutrition status (*n* = 1), and nutrition screening (*n* = 1); 1 unpublished thesis incorporated multiple concepts. Few randomised clinical trials that quantify aspects of nutrition management for critically ill patients requiring NIV have been conducted. Further studies, particularly those focusing on the impact of nutrition during NIV on clinical outcomes, are required to inform clinical practice.

## 1. Introduction

Nutrition is an integral component of therapy in the intensive care unit (ICU). While clinical practice guidelines generally recommend oral intake as the preferred route for nutrition during critical illness, the majority of targeted recommendations on nutrition practice relate to enteral nutrition (EN) support [1,2]. In addition, where peer-reviewed literature does exist, it has primarily been undertaken in patients receiving invasive mechanical ventilation (IMV).

IMV is the respiratory support provided by the partial or complete replacement of normal breathing via the insertion of a tube into the airway. It typically requires sedation and is associated with complications that can lead to an extended hospital admission including infection, pneumonia, or pneumothorax [3,4]. EN is the preferred route of nutrition delivery for patients expected to require more than 48 h of IMV, a recommendation influenced by the need for sedation and the presence of the breathing tube preventing oral intake.

Respiratory support can also be provided in the ICU via non-invasive techniques. Non-invasive ventilation (NIV) provides respiratory support via an external interface, such as a helmet or mask, and its use has increased substantially in the past two decades (6.92% in 2009, 9.64% in 2013 (relative risk 1.07 (95% confidence interval: 1.06–1.08)), *p* < 0.001) [5]. This trend has occurred across a period where illness severity of ICU admissions has also increased [5], dismissing the notion that those who require NIV are less unwell than those requiring IMV. Despite this growing patient population, critical care guidelines provide limited recommendations on nutrition provision and management during NIV.

The use of NIV poses several barriers to nutrition delivery. Patients may be fasted if intubation is anticipated [6]. Provision of oral intake may require removal of the NIV interface, and the position of an enteral feeding tube can cause an air leak, which may impact respiratory function [7]. While parenteral nutrition (PN) is an alternative, it is more costly, poses an infection risk [8,9], and is typically reserved for patients experiencing gastrointestinal failure.

To ensure optimal nutrition management of the growing cohort of patients receiving NIV, it is essential to first understand what literature exists on this topic. The objective of this scoping review was to identify original research related to the nutrition management of critically ill adult patients requiring NIV and the key concepts addressed. Sub-questions regarding concepts of nutrition management were also explored, including: (1) what is the primary route of nutrition; (2) what methods are used to determine macronutrient requirements; (3) are calorie and protein intakes adequate compared to estimated requirements; and (4) are there barriers to adequate nutrition provision?

## 2. Materials and Methods

### 2.1. Search Strategy

A scoping review of the literature was conducted in accordance with the JBI Manual for Evidence Synthesis [10] and the protocol published prior to commencement [11]. Following a preliminary search of the online database Medical Literature Analysis and Retrieval System Online (MEDLINE) via Ovid SP to ascertain common keywords, a search strategy was developed to identify original research papers across three strings: (1) critically ill adults; (2) NIV; and (3) nutrition. The final MEDLINE search (Table 1) was adapted for Excerpta Medica Database (Embase) and Scopus via Elsevier, Web of Science, and Google Scholar (Supplemental Tables S1–S4) and was conducted on the 17 November 2021.

### 2.2. Trial Selection

Search results were exported to Endnote reference manager software (Clarivate Analytics, Version 20) and duplicates removed before uploading the remaining articles to Covidence (Veritas Health Innovation, Melbourne, Australia). Two authors (E.VS and L.S.C) independently conducted a title and abstract review against eligibility criteria and retrieved full texts for potentially relevant articles. The full texts were screened in Covidence in duplicate (E.VS and L.S.C) for inclusion in the final review, with discrepancies resolved through discussion.

Articles were included if they: (1) were conducted in adult patients (as defined by the paper or ≥16 years of age) recruited in an ICU and receiving NIV (excluding low- and high-flow nasal cannula or IMV); (2) addressed an aspect of nutrition management; and (3) were made available from 1990 onwards in the English language. Articles were excluded if they were review articles (systematic, literature, or integrated reviews), case studies, or opinion pieces.

### 2.3. Data Extraction

Data were extracted from the final articles included in the scoping review using the pre-defined data-extraction tool (Supplemental Table S5). Data extraction included specific details about the aim, study design, geographic location, number of sites, the population (number of patients, age, sex), context (type of ICU, NIV use, duration, and interface) and nutrition concept studied (route of nutrition, nutrition intake/adequacy, method for determining nutrition requirements, or other nutrition management themes identified during the review), and outcomes related to the scoping review question.

## 3. Results

### 3.1. Search Results

**Figure 1:**
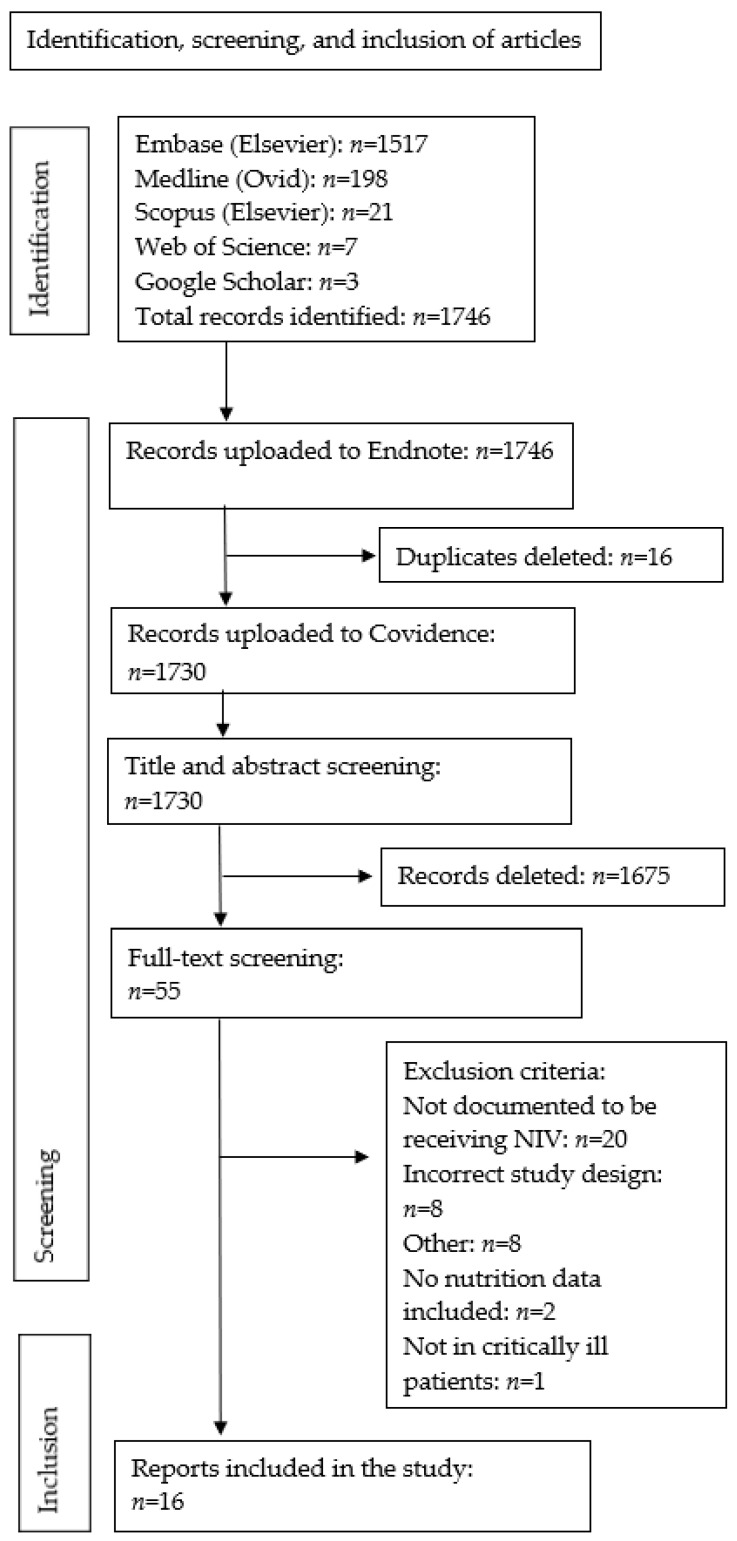
Flow chart for the identification, screening and inclusion of studies.

The online database and grey literature searches identified 1746 articles. Sixteen duplicates were removed in Endnote, leaving 1730 articles to be uploaded to Covidence. Full-text screening was completed on 55 articles, and 16 were included in this scoping review (exclusion reasons provided in Figure 1).

### 3.2. Study Characteristics

Study characteristics are presented in Table 2. The 16 included studies were published between 1999 and 2021 and included 9 full-text publications [12,13,14,15,16,17,18,19,20] and 7 abstracts [21,22,23,24,25,26,27]. The majority of the studies (*n* = 14) were prospective in nature [12,13,14,15,17,18,20,21,22,23,24,25,26,27], with 2 being retrospective [16,19]. More than half of the studies (*n* = 10) were observational in design [12,13,14,16,17,18,19,21,22,23], with 6 interventional studies [15,20,24,25,26,27] (Table 2). Fifteen were single-centre studies [12,13,14,15,16,17,18,20,21,22,23,24,25,26,27], with the sole multi-centre study covering 20 ICUs from a single country [19].

### 3.3. Population

The median number of participants for the 16 studies was 34. Fifteen of the studies included <107 patients [12,13,14,15,16,17,18,20,21,22,23,24,25,26,27], with only 1 large cohort of 1075 patients [19]. Seven studies were conducted in patients with respiratory related diagnoses [16,17,18,20,22,23,25], 5 were conducted in patients with mixed diagnoses [12,14,15,19,27], 2 did not define the diagnosis [13,21], and 1 was in patients post liver transplantation [24]. The country that produced the largest number of studies was Australia (*n* = 5) [12,13,14,17,18].

### 3.4. Context

All 16 studies were conducted in an ICU, with 3 also including patients from specialist respiratory wards [16,18,22] (Table 3). Nine studies reported the use of NIV in their study to be pre-intubation (*n* = 5) [12,16,18,19,24], post-extubation (*n* = 2) [15,27], or both (*n* = 2) [14,17]. The use or purpose of NIV was not clear in the remaining 7 studies [13,20,21,22,23,25,26]. Six studies did not specify the length of time patients received NIV [12,13,19,20,22,26], while 7 studies reported length of time on NIV in days [15,16,18,21,23,25,27] and 3 in hours [14,17,24]. Eight studies did not report the interface used to deliver NIV [13,18,20,21,22,23,24,27]. For the 8 studies that reported the interface, 6 used more than one type (face mask with modification, nose mask, oro-nasal mask, or helmet) [12,15,17,19,25,26]. Nine studies did not specify mode or parameters of NIV delivered [12,13,18,19,21,22,23,25,26], and those that did reported a combination of bilevel and/or continuous positive airway pressure support, spontaneous/timed ventilation, and pressure support ventilation [14,15,16,17,20,24,27].

### 3.5. Concepts

Seven of the studies focused on the concept of route of nutrition [16,17,19,20,23,25,26], 4 on quantifying nutrition intake [12,18,21,24], 2 on resting energy expenditure [15,27], 1 each on nutrition status [22] and nutrition screening [13], and 1 unpublished thesis focused on nutrition practice, incorporating multiple concepts [14].

#### 3.5.1. Route of Nutrition

Eight studies reported on route of nutrition: 3 on its association with clinical outcomes [16,19,20], 2 observational studies on the route received [23,26], 2 on implications of the presence of a naso-enteric tube [17,25], and 1 on the association of route and nutrition adequacy [14]. Amongst these studies, there was no consensus on the preferred route of nutrition. Two studies reported rates from each of the 4 main routes—no, oral, enteral, or parenteral nutrition—in which oral was most common in 1 (*n* = 20 of 30 patients) [14], and no nutrition was most common in the other (*n* = 622/1075) [19]. In this larger, multi-centre study, PN was also more common than EN [19]. Two studies reported on the number of patients receiving oral, enteral, or a combination of these but not on rates of no nutrition or PN [23,26]. One reported oral intake as the most common route (*n* = 18/32) [23] and the other oral combined with naso-enteric feeding (*n* = 20/39) although the groups in this second study were not entirely clear [26].

Three studies compared route of nutrition and clinical outcomes. Each of these 3 studies compared different routes of nutrition and different outcomes. Terzi et al. reported a multivariate analysis of 4 nutrition groups (no, oral, enteral, or parenteral nutrition) [19], and Kogo et al. compared EN with non-enteral nutrition (defined as those not receiving EN or volitional intake but fluid replacement or PN) [16]. Although different outcomes were assessed, both studies reported associations with route of nutrition. One intervention study investigated the use of standardised EN compared to oral nutrition, reporting improved nutrition status and immunological functioning in patients who received the standardised EN therapy [20]; however, a definition of nutrition status was not included.

Two studies reported on the use of naso-enteric feeding tubes: 1 on outcomes (enteric drainage/feeding, patient comfort, and air leaks) when using a naso-enteric tube with an investigator modified face mask [25] and 1 on the impact the presence of a naso-enteric tube had on the success or failure of NIV [17].

Jeong et al. reported on the number of patients in their study cohort receiving different routes of nutrition and the association of route with intake adequacy. This thesis grouped intake as <50% or ≥50% estimated requirements and reported no significant association with route and meeting 50% estimated energy requirements (EER) and PN being associated with consuming 50% estimated protein requirements (EPR) [14].

#### 3.5.2. Nutrition Intake

Five studies reported on calorie and/or protein intake as a nutrition concept [12,14,18,21,24]: 2 reported on calorie intake only, concluding that patients receiving NIV in ICU consume <1000 kilocalories (kcal)/day [21] and 52% less than those receiving high-flow nasal cannula therapy [24]. Three studies reported on calorie and protein intakes [12,14,18], all concluding that nutrition intake (calorie and protein) was inadequate (compared to estimated requirements) during NIV use. Jeong et al. reported no difference in nutrition adequacy between different modes of NIV (Bilevel Positive Airway Pressure or Continuous Positive Airway Pressure) [14], while Chapple et al. reported an increase in nutrition intake with decreasing respiratory support (NIV vs. nasal cannula vs. no oxygen therapy) [12].

#### 3.5.3. Resting Energy Expenditure

Two studies reported on resting energy expenditure (REE) during different modes of NIV [15,27]. Kilger et al. [15], using Deltatrac MBM-100 (Datex, Helsinki, Finland), reported non-invasive positive pressure ventilation reduced REE during continuous positive airway pressure and even further during pressure support ventilation compared to spontaneous breathing (Table 3). Similarly, Steele [27] reported “statistically significant beneficial changes in resting energy expenditure (REE) during NPPV when compared with spontaneous ventilation with CPAP” although no further details were provided.

#### 3.5.4. Nutrition Screening

One study reported on the feasibility of completing nutrition screening tools for non-invasively mechanically ventilated patients [13], stating that the Malnutrition Universal Screening Tool (MUST) was quicker to complete than the modified Nutrition Risk in Critically Ill (mNUTRIC) (Table 3). Barriers to completion of the MUST included obtaining current and previous weight and limited availability of family to provide collateral history. Barriers for the mNUTRIC included staff training, time taken to obtain data, and a lack of automation in calculating the score.

#### 3.5.5. Nutrition Status

One study assessed the impact of nutrition status on NIV [22], concluding they were related (*p* < 0.01), but the direction of the relationship or the tool used to determine nutrition status were not stated.

## 4. Discussion

This is the first scoping review to identify current literature relating to the nutrition management of critically ill adult patients requiring NIV. The literature on this topic is limited, with just 16 studies identified from the last 30 years. Studies were predominately of a single centre, observational nature with small participant numbers (most recruited <100 patients), and nearly half were reported in abstract form only. They largely comprised lower-quality (level III) evidence, with a small number of level II (randomised control trials (RCT)) and no level I (systematic review) literature. This differs substantially from ICU nutrition literature outside of this review, in which at least 9 RCTs [28,29,30,31,32,33,34,35,36], 6 enrolling large cohorts (>1000 patients) [28,30,32,33,35,36], have been published in the last 10 years. While these have predominately involved patients receiving IMV, they addressed key nutrition management questions, such as optimal route [29,33,35] and timing of nutrition [28], adequacy of nutrition and associated outcomes [30,31,34,36], in addition to the use of supplemental glutamine and anti-oxidants [32]. This higher-quality evidence over a broad range of nutrition management topics demonstrates the stark difference in the quality of literature pertaining to patients receiving NIV.

The key concept identified in this review was the route of nutrition during NIV. While no optimal route of feeding was reported, “nil nutrition” or oral nutrition were reported more frequently than EN or PN. The majority of studies were of a single-centre, observational nature and hence likely reflect site-specific practice more than the nutrition route with greatest clinical benefit. In other non-mechanically ventilated critically ill populations, oral intake in isolation has been demonstrated to be inadequate to meet nutrition requirements. In the first 7–14 days post-extubation, patients that progress to oral intake meet <50% of EER [37,38]. ICU survivors within the post-ICU acute ward setting also experience inadequate oral nutrition, meeting 37% (interquartile range (IQR) 21–67%) of predicted energy requirements [39]. In this same population, adequate nutrition was best achieved when oral intake was supplemented with EN (104% (IQR 66–132%) of predicted energy requirements) [39]. This is similar for the sub-population of traumatic brain injury survivors, whose caloric intake was nearly halved when consuming oral intake compared to EN [40]. Comparisons of EN and PN in the NIV population are limited, but in predominately IMV populations, delivery of EN encounters a number of barriers [41], which can be mitigated by the use of intravenous PN. Although PN has also been reported in a meta-analysis to be associated with higher infection rates compared to EN [9], this association was most notable in the RCTs where PN provided increased calories compared to EN, with no effect seen in RCTs where calorie provision was similar between the 2 routes [9]. When considering what the optimal nutrition route is, adequate intake is not the sole factor, and both clinical risk and patient outcome must also be considered. Therefore, robust evidence building on the studies reported here are required to understand the route of nutrition delivery that provides optimal benefit for these patients in terms of both intake and clinical outcomes.

Five studies explored the concept of the amount of nutrition consumed. Across these, there was a clear consensus that nutrition intake was low and when compared to estimated requirements was inadequate. Mean intake was reported at 37–42% of EER and 28–35% of EPR [12,14]. This is less compared to patients receiving IMV in the ICU who have, across the globe, received 50–60% EER (EN, PN, and propofol) and 52% EPR [42] although some of this difference in energy provision may be accounted for in propofol use during IMV. Understanding nutrition intake and adequacy in NIV patients is important, as previous research has shown that differences in caloric provision for IMV patients have not impacted short- or long-term outcomes when delivered early in the ICU stay [30,36,43]. Rice et al. [30] compared trophic feeding (25% of calculated caloric goal or 400 kcal/day) to full feeding (80% of calculated caloric goal or 1300 kcal/day), while Chapman et al. [36] compared energy-dense (1.5 kcal/mL providing 1863 kcal/day) to routine enteral nutrition (1.0 kcal/mL providing 1262 kcal/day). For both these studies, the length of the nutrition intervention was 6 days compared to predominately 2–5 days in the studies included here. However, no studies in this review addressed both nutrition adequacy and clinical outcomes.

Resting energy expenditure was addressed as a nutrition concept in 2 studies, both of which focused on the difference in expenditure between different modes of NIV support. A limitation of both studies is the lack of clarity regarding the methodology for measuring energy expenditure. Neither study detailed whether indirect calorimetry was used for air capture and, if so, how this was achieved on a NIV circuit or which equation was used if the REE was calculated from VO_2_ and VCO_2_ measurements. This knowledge is imperative because clinical practice guidelines recommend the use of indirect calorimetry as the gold standard for determining energy expenditure, but publications in healthy [44] and non-ICU [45] populations have highlighted both methodological and clinical practice concerns with its use during NIV. Indirect calorimetry is reliant on both inspired and expired air capture [46], which is more accurately undertaken with a closed-loop respiratory circuit during IMV than an open circuit, as with NIV. None of the included studies explored or compared the use of alternate methods for estimating or measuring energy expenditure in patients receiving NIV, highlighting the lack of development of such assessments in this population and inadequacy of knowledge regarding which energy levels should be targeted.

Nutrition screening and status are concepts identified in the included literature. Both the study of malnutrition screening tools [13] and the use of anthropometric measurements to incorporate in nutrition assessments [12] were feasibility studies. Neither addressed validity or drew conclusions about which screening or assessment tools should be used in the NIV population. However, this is consistent with evidence for patients receiving IMV, for whom a nutrition screening or assessment tool that is valid, clinically feasible, and supported by robust evidence has not been widely adopted or endorsed by clinical practice guidelines [1,2,47]. Screening “at-risk” patients is important for preventing hospital acquired malnutrition, and nutrition assessment aims to identify and diagnose malnutrition in order to facilitate targeted nutrition care [47]. Although different in concept, they have a shared goal of mitigating the negative impact malnutrition may have on mortality, infection, and length of stay [48].

A strength of this scoping review is the rigorous methodology employed, including collaboration with a librarian to develop the keyword search and independent screening and data extraction by two of the authors. Furthermore, all authors are clinician-researchers with a good understanding of the translation of research studies to clinical practice. This review was limited to literature published in English. Conclusions and comparisons are limited by the diverse methodology for collecting and reporting on both nutrition and NIV; inherent differences in methods to quantify nutrition intake or requirements means comparisons should be made with caution. The phrase “non-invasive ventilation” is used in the literature both broadly to capture all patients in ICU who are not invasively mechanically ventilated and more specifically to define those receiving mechanical ventilation through a non-invasive interface. Developing consensus between these definitions is important for contextualising the impact on nutrition support.

## 5. Conclusions

Sixteen studies addressing nutrition concepts in patients receiving NIV in ICU were identified, of which the majority were single-centre and observational studies. The key concept identified was the route of nutrition; yet, there was a lack of consensus regarding the preferred route for clinical benefit. Studies addressing calorie and protein intake concluded these were inadequate compared to estimated requirements. The literature has few randomised controlled trials and lacks studies that address nutrition-related outcomes following NIV in ICU, which impedes the development of clinical practice recommendations.

## Figures and Tables

**Table 1:** MEDLINE (Ovid) Search Strategy.

**No.**	**Search** (mp = title, abstract, original title, name of substance word, subject heading word, floating sub-heading word, keyword heading word, organism supplementary concept word, protocol supplementary concept word, rare disease supplementary concept word, unique identifier, synonyms)
1	(critical ill*) or (critical care) or (intensive care unit*) or (respiratory care unit*) or (critical adj care) or (intensive adj1 care adj1 unit*)
2	(noninvasive ventilation) or (artificial respiration) or (respiratory insufficiency) or (positive pressure respiration) or (continuous positive airway pressure) or (intermittent positive airway pressure) or (respiratory distress syndrome) or (noninvasive adj2 ventilation)
3	(energy intake) or (enteral nutrition) or (parenteral nutrition) or (total parenteral nutrition) or (nutrition assessment) or (nutrition* status) or (nutrition support) or (eating) or (nutrition management) or (indirect calorimetry) or (basal metabolism) or (resting energy expenditure) or (oral intake)
4	1 AND 2 AND 3
5	(child* or infan* or pediatr* or paediatr* or neonat* or preterm or newborn*)
6	4 NOT 5
7	Limit 6 to English language
8	Limit 7 to yr = “1990–Current”

* represents a truncation command for searching in the MEDLINE (Ovid) database.

**Table 2:** Aim, study design, and population of included studies.

First Author, Publication Year	Geographic Location	Format	Aim	Study Design	Population: Number, Age, Sex
Arnaout, 2015 [21]	France	Abstract	To evaluate caloric intakes of pts receiving NIV irrespective of the indication for NIV	Prospective, observational, single centre	*n* = 90, 73 (64, 91) ^a^ y, 45 M
Biswas, 2019 [22]	Bangladesh	Abstract	To investigate the possible effect of NIV on outcomes (demographics, aetiology of a HRF episode, co-morbidities, biochemical parameters)	Prospective, observational, single centre	*n* = 102, age NS, sex NS
Chapple, 2020 [12]	Australia	Full text	To quantify intake and nutrition-related outcomes of non-IMV critically ill patients and to establish feasibility of methods to measure nutrition-related outcomes in this population	Prospective, observational, single centre	*n* = 20, 53 (42, 64) ^a^ y, 10 M
Digby, 2012 [23]	Canada	Abstract	To describe the use of enteral nutrition, pharmacological prophylaxis of stress ulcers, and VTE in critically ill patients receiving NIV	Prospective, observational, single centre	*n* = 32, 71.5 ± 15.7 ^b^ y, 12 M
Egan, 2021 [13]	Australia	Full text	To compare feasibility of MUST vs. mNUTRIC for identifying non-invasively mechanically ventilated pts with nutritional or malnutrition risk	Prospective, observational, feasibility, single centre	*n* = 20, 65.3 ± 13.9 ^b^ y, 12 M
Gupta, 2016 [24]	India	Abstract	To compare HFNC vs. NIV as the modality to manage ARF in postoperative hypoxemia in post-liver-transplant patients	Prospective, single-centre, pilot RCT	*n* = 20 (10 per group), age NS, sex NS
Jeong, 2017 [14]	Australia	Thesis	To explore current nutritional care practice provided to the patients receiving NIPPV therapy in ICU	Prospective, observational, single centre	*n* = 30, 62.6 ± 14.0 ^b^ y, 14 M
Kilger, 1999 [15]	Germany	Full text	To investigate the effects of NIPPV on pulmonary gas exchange, breathing pattern, intrapulmonary shunt fraction, oxygen consumption, and resting energy expenditure in patients with persistent ARF but without COPD after early extubation	Prospective, interventional, single centre	*n* = 15, 47 ± 12 ^b^ y, 8 M
Kogo, 2017 [16]	Japan	Full text	To determine whether administration of EN to subjects receiving NIV would increase airway complications and worsen outcomes by causing severe hypoxia and/or pneumonia	Retrospective, cohort, single centre	*n* = 107 EN: *n* = 60 pts, 77 (68, 83) ^a^ y, 47 M Non-EN: *n* = 47, 73 (64, 81) ^a^ y, 33 M
Korula, 2020 [17]	Australia	Full text	To evaluate NIV failure rate and factors associated with NIV failure	Prospective, observational, single centre	*n* = 60, 62 ± 17.6 ^b^ y, 34 M
Minev, 2015 [25]	Bulgaria	Abstract	Not clearly defined	Prospective, interventional, single centre	*n* = 6, age NS, sex NS
Pearson, 2017 [26]	United States	Abstract	To determine the rate of enteral nutrition in patients with ARDS receiving NIV with a helmet strategy compared to face mask	Prospective, single centre, RCT	*n* = 83, age NS, sex NS
Reeves, 2014 [18]	Australia	Full text	To measure energy and protein intakes of patients in acute respiratory failure requiring NIV	Prospective, observational, single centre	*n* = 36, 65 ± 9 ^b^ y, 12 M
Steele, 2000 [27]	Not specified	Abstract	To examine the effect of NIPPV on pulmonary gas exchange, breathing pattern, intrapulmonary shunt fraction, oxygen consumption, resting energy expenditure, and weaning success	Prospective, interventional (patient own controls), single centre	*n* = 15, age NS, sex NS
Terzi, 2017 [19]	France	Full text	To describe the nutritional management of patients starting first-line NIV	Retrospective, cohort, multi-centre	*n* = 1075, demographics by route of nutrition: NoN: 70.4 (59.4, 80.2) ^a^ y, 384 M PN: 67.3 (56.4, 78.8) ^a^ y, 47 M EN: 66.6 (60.9, 77.3) ^a^ y, 19 M ON: 71.6 (59.4, 80.3) ^a^ y, 206 M
Zhang, 2021 [20]	China	Full text	To investigate the effects of standardised EN on nutritional indicators and immunological functioning of acute exacerbations of COPD patients with respiratory failure	Prospective, single centre, RCT	*n* = 92 (46 per group), Control: 67.46 ± 5.21 ^b^ y, 29 M; Observation: 68.55 ± 5.39 ^b^ y, 27 M

^a^ Median (Interquartile range), ^b^ Mean ± Standard Deviation. ARDS, acute respiratory distress syndrome; ARF, acute respiratory failure; COPD, chronic obstructive pulmonary disease; EN, enteral nutrition; HFNC, high-flow nasal cannula; HRF, hypoxic respiratory failure; ICU, intensive care unit; M, male; mNUTRIC, modified Nutrition Risk in Critically Ill; MUST, malnutrition universal screening tool; *n*, number; NIPPV, non-invasive positive pressure ventilation; NIV, non-invasive ventilation; NoN, no nutrition; non-EN, non-enteral nutrition; non-IMV, non-invasively mechanically ventilated; NS, not specified; ON, oral nutrition; PN, parenteral nutrition; pts, patients; RCT, randomised control trial; VTE, venous thromboembolism; y, years.

**Table 3:** Concept, context, and relevant results of included studies.

First Author, Publication Year	Concept	Context: Type of ICU, Use of NIV, Length of NIV, NIV Interface	Outcomes/Relevant Results
Arnaout, 2015 [21]	Caloric intake	MICU, use of NIV NS, 5 days reported, NS	Majority of patients received <1000 kcal/day (results per day for the first 5 days).
Biswas, 2019 [22]	Nutrition status	Respiratory care unit and ICU, use of NIV NS, length of NIV NS, NIV interface NS	Nutrition status is associated with NIV outcomes (*p* < 0.001). NB: nutrition status definition not reported.
Chapple, 2020 [12]	Calorie and protein intake	Mixed ICU, NIV used pre-intubation, length of NIV NS, face mask, oro-nasal mask	Median energy and protein intake per meal of patients receiving NIV (face mask and oro-nasal mask): 278 (0, 1404) ^a^ kJ and 1.2 (0, 8.0) ^a^ g protein (per meal across 3 consecutive study days, *n* meals = 12).
Digby, 2012 [23]	Route of nutrition	Type of ICU not specified, use of NIV NS, 2.53 ± 1.76 ^b^ days, NIV interface NS	78.1% (*n* = 25) received enteric nutrition (feeding tube, oral intake, or combination) after 24 h of NIV; for 68.8% (*n* = 22) of these patients, EN continued until NIV was discontinued (2.41 ± 1.8 ^b^ days). Oral route was most common (*n* = 18).
Egan, 2021 [13]	Nutrition screening	Mixed ICU, use of NIV NS, length of NIV NS, NIV interface NS	MUST = 8.1 ± 2.8 ^b^ (range 4–14) min; mNUTRIC = 22 ± 5.6 ^b^ (range 13–33) min.
Gupta, 2016 [24]	Caloric intake	Liver transplant ICU, NIV used pre-intubation, 48 h, NIV interface NS	All patients were fed either oral or enteral nutrition, but the NIV group consumed 52% less calories compared to patients receiving HFNC. This was largely due to the inability to feed orally and apprehension of aspiration due to aerophagia when fed enterally.
Jeong, 2017 [14]	Multiple concepts, including route of nutrition, calorie and protein intake and adequacy	Mixed ICU, NIV used pre-intubation and post-extubation, 45.1 ± 47.5 ^b^ (range 6–235) hours, oro-nasal mask	67% received ON, 10% nil nutrition, 7% EN, 7% PN, 7% thickened fluids only, 3% Oral + EN. Energy intake: 2277 ± 1776 ^b^ kJ/d, 70% failed to meet 50% EER. Protein intake: 29 ± 32 ^b^ g/d, 83% of pts failed to meet 50% of EPR. Number of study days not reported.
Kilger, 1999 [15]	Resting energy expenditure	Mixed ICU, NIV used post-extubation, 2 (range 1–20) days, face mask, nose mask	NIPPV reduced REE during CPAP (1454 ± 204 ^b^ kcal/day) and even further during PSV (1332 ± 234 ^b^ kcal/day) compared to SPB (1658 ± 220 ^b^ kcal/day)
Kogo, 2017 [16]	Route of nutrition	ICU and respiratory ward, NIV used pre-intubation, non-EN: 8 (5, 20) ^a^ days, EN: 16 (7, 43) ^a^ days, face mask	Rates of mucus plug (50% vs. 30%), aspiration pneumonia (17% vs. 4%), airway complication (53% vs. 32%) were higher in the EN group than non-EN group. Survivors in the EN group stayed longer in the ICU (14 (5,25) ^a^ days) and were less likely to be discharged home (36%) compared to the non-EN group (7 (3,17) ^a^ and 8%).
Korula, 2020 [17]	Route of nutrition	Mixed ICU, NIV used pre-intubation and post-extubation, 25.5 (6.7, 69.4) ^a^ hours, face mask oro-nasal mask, helmet	The NGT was placed or was present in situ at the commencement of NIV in 34 of 70 episodes (13 primary, 21 secondary), but EN was administered in only 20 of those who had NGT (28.5%). Episodes of NIV in which the patient had an NGT in situ had higher odds of NIV failure (odds ratio 6.2 (1.9, 19.8); *p* < 0.01).
Minev, 2015 [25]	Route of nutrition	ICU, use of NIV NS, 3.5 ± 1.6 ^b^ days, face mask (standard and modified by authors)	The investigator modified mask achieved adequate drainage of the stomach and/or enteral nutrition, with improved comfort and no additional air leaks.
Pearson, 2017 [26]	Route of nutrition	Medical ICU, use of NIV NS, length of NIV NS, face mask, helmet	EN + face mask: *n* = 16 (41%), EN + helmet: *n* = 27 (61.4%) (*p* = 0.06), ON + face mask: *n* = 12 (31%), ON + helmet: *n* = 22 (50%) (*p* = 0.08).
Reeves, 2014 [18]	Calorie and protein intake	ICU and respiratory ward, NIV used pre-intubation, 4.7 ± 7.0 ^b^ days, NIV interface NS	Energy and protein intakes were 1434 ± 627 ^b^ kcal + 63 ± 29 ^b^ g protein (across 283 study days). 75% patients consumed <80% of energy and protein requirements.
Steele, 2000 [27]	Resting energy expenditure	ICU, NIV used post-extubation, 2 days (no IQR or range provided), NIV interface NS	Statistically significant beneficial changes in REE during NIPPV when compared with SPB with CPAP.
Terzi, 2017 [19]	Route of nutrition	Multiple ICU, NIV used pre-intubation, length of NIV NS, face mask, nasal mask	Most patients (*n* = 622 (57.9%)) received no nutrition during the first 2 days of NIV, with *n* = 351 (32.7%) receiving ON, *n* = 74 (6.9%) receiving PN, and *n* = 28 (2.6%) receiving EN.
Zhang, 2021 [20]	Route of nutrition	Type of ICU NS, use of NIV NS, length of NIV NS, NIV interface NS	Hb, serum albumin and serum total protein were not different between the two groups at baseline but increased from pre to post treatment, with the observational group having higher post-treatment indicators than control group (*p* < 0.05).

^a^ Median (Interquartile range), ^b^ Mean ± Standard Deviation. CPAP, continuous positive airway pressure; EER, estimated energy requirements; EN, enteral nutrition; EPR, estimated protein requirements; g, gram; Hb, haemoglobin; HFNC, high-flow nasal cannula; ICU, intensive care unit; IQR, interquartile range; kcal, kilocalorie; kJ, kilojoule; MICU, expansion of abbreviation not provided in original article; mNUTRIC, modified Nutrition Risk in Critically Ill; MUST, malnutrition universal screening tool; *n*, number; NB, note; NGT, nasogastric tube; NIPPV, non-invasive positive pressure ventilation; NIV, non-invasive ventilation; non-EN, non-enteral nutrition; NS, not specified; ON, oral nutrition; PN, parenteral nutrition; PSV, pressure support ventilation; pts, patients; REE, resting energy expenditure; SPB, spontaneous breathing.

## Data Availability

No new data were created or analyzed in this study. Data sharing is not applicable to this article.

## Supplementary Materials

The following supporting information can be downloaded at: https://www.mdpi.com/article/10.3390/nu14071446/s1, Table S1: ELSEVIER—Embase Search Strategy; Table S2: ELSEVIER—Scopus Search Strategy; Table S3: Web of Science Search Strategy; Table S4: Google Scholar Search Strategy; Table S5: Data-Extraction Tool.
