# Supplementary material for: Nutrition Management for Critically Ill Adult Patients Requiring Non-Invasive Ventilation: A Scoping Review
Source: Nutrients. 2022 Mar 30;14(7):1446. doi: 10.3390/nu14071446 (PMC9003108; doi:10.3390/nu14071446)
Supplement: Supplementary file 1 [file nutrients-14-01446-s001.zip › nutrients-1637567-supplementary.pdf]

Supplemental Table S1: ELSEVIER – Embase Search Strategy

No.	**Search** (untagged=all searchable fields which includes titles, abstracts, index terms)	**Records Retrieved**
**1**	(critically ill patient) or (critical illness) or (intensive care) or (intensive care unit) or (intensive care patient) or (critical NEXT/1 care) or (critical NEAR/1 care) or (intensive NEXT/1 care) or (intensive NEAR/1 care) or (intensive NEXT/1 care NEXT/1 unit) or (intensive NEAR/1 care NEAR/1 unit) or (intensive NEAR/1 care NEAR/1 unit) or (intensive NEXT/1 care NEXT/1 units) or (intensive NEAR/1 care NEAR/1 units)	1,469,100
**2**	(noninvasive ventilation) or (artificial ventilation) or (respiratory failure) or (positive pressure ventilation) or (continuous positive airway pressure) or (intermittent positive airway pressure) or (respiratory distress syndrome) or (noninvasive NEXT/2 ventilation) or (noninvasive NEAR/2 ventilation)	425,856
**3**	(caloric intake) or (enteric feeding) or (nutrition supplement) or (parenteral nutrition) or (parenteral solution) or (total parenteral nutrition) or (total parenteral solution) or (peripheral parenteral nutrition) or (nutrition assessment) or (nutrition status) or (protein calorie malnutrition) or (indirect calorimetry) or (indirect calorimeter device) or (basal metabolic rate) or (resting energy expenditure) or (oral intake)	467,952
**4**	#1 AND #2 AND #3	6110
**5**	child* or infan* or pediatr* or paediatr* or neonat* or preterm or newborn*	5,089,581
**6**	#4 NOT #5	4025
**7**	#4 NOT #5 AND [english]/lim	3790
**8**	#7 NOT [medline]/lim	1529
**9**	#8 AND [1990-2021]/py	1517

Supplemental Table S2: ELSEVIER – Scopus Search Strategy

No.	**Search** (within article title, abstract, key words)	**Records Retrieved**
1	nutrition	477,332
2	critical care	217,737
3	non-invasive ventilation	8966
4	#1 AND #2 AND #3	21
5	# 4 AND PUBYEAR >1989	21

Supplemental Table S3: Web of Science Search Strategy

No.	**Search** (topic=title, abstract, author keywords, and Keywords Plus)	**Records Retrieved**
1	nutrition	293,273
2	critical care	116,004
3	non-invasive ventilation	6746
4	1 AND 2 AND 3	7
5	4 (limited to publication 1990-01-01 to 2021-11-17)	7

Supplemental Table S4: Google Scholar Search Strategy

No.	Search	Records Retrieved
1	nutrition AND critical care AND non-invasive ventilation	0
2	nutrition AND intensive care AND non-invasive ventilation	1 additional abstract<br>1 additional thesis submission<br>1 additional research paper

Supplemental Table S5: Data Extraction Tool

**Data Extraction – Article #**		
**Evidence Source Details and Characteristics**		
Citation details	Authors	
	Date	
	Title	
	Journal	
	Volume	
	Issue	
	Pages	
Country/s of origin		
Aim/objective/hypothesis that this study addresses		
Study design		
**Details extracted from source of evidence**		
Participants (intensive care)		
From methods	Inclusion criteria	
	Exclusion criteria	
From results	Number	
	Age	
	Sex	
	ICU admission diagnosis	
	Length of ICU admission	
	Other (e.g. mortality)	
Concept (nutrition management)		
	Route of nutrition	
	Method for determining nutrition requirements	
	Calorie intake/adequacy	
	Protein intake/adequacy	
	Barriers to nutrition provision (oral, enteral, parenteral)	
	Strategies for nutrition management	
	Other	
Context (non-invasive ventilation)		
	Number and type of ICU	
	Length of NIV	

	Type of apparatus used to deliver NIV	
	Type of NIV delivered	
	Other	
Primary outcome		
Key findings that relate to the scoping review question/s		
